# Development and user testing of a patient decision aid for cancer patients considering treatment for anxiety or depression

**DOI:** 10.1186/s12911-023-02146-y

**Published:** 2023-04-06

**Authors:** Rebecca Rayner, Joanne Shaw, Caroline Hunt

**Affiliations:** 1 School of Psychology, Faculty of Science, The University of Sydney, Chris O’Brien Lifehouse Level 6 (North), C39Z, 2006 Sydney, NSW Australia; 2 Psycho-oncology Co-operative Research Group, School of Psychology, Faculty of Science, The University of Sydney, Sydney, NSW Australia

**Keywords:** Anxiety, cancer, Cognitive interviews, Decision aids, Depression, Oncology, Psycho-oncology, Qualitative, Treatment

## Abstract

**Background:**

Despite high rates of mental health disorders among cancer patients, uptake of referral to psycho-oncology services remains low. This study aims to develop and seek clinician and patient feedback on a patient decision aid (PDA) for cancer patients making decisions about treatment for anxiety and/or depression.

**Methods:**

Development was informed by the International Patient Decision Aid Standards and the Ottawa Decision Support Framework. Psycho-oncology professionals provided feedback on the clinical accuracy, acceptability, and usability of a prototype PDA. Cognitive interviews with 21 cancer patients/survivors assessed comprehensibility, acceptability, and usefulness. Interviews were thematically analysed using Framework Analysis.

**Results:**

Clinicians and patients strongly endorsed the PDA. Clinicians suggested minor amendments to improve clarity and increase engagement. Patient feedback focused on clarifying the purpose of the PDA and improving the clarity of the values clarification exercises (VCEs).

**Conclusions:**

The PDA, the first of its kind for psycho-oncology, was acceptable to clinicians and patients. Valuable feedback was obtained for the revision of the PDA and VCEs.

**Supplementary Information:**

The online version contains supplementary material available at 10.1186/s12911-023-02146-y.

## Background

Approximately one-third of cancer patients meet diagnostic criteria for one or more mental health disorders [1] and cancer survivors report high rates of anxiety and depression up to ten years post-treatment [2]. The implications of untreated mental health disorders among cancer patients include longer hospitalisation [3], poorer survival prognosis [4], and increased risk of suicide [5]. Effective treatments are available. Medium to large effects of psychological interventions are reported among patients with clinical levels of anxiety and depression, sustained 6 to 12 months post-intervention [6]. Pharmacologic treatments are also effective. Despite limited studies in cancer patients specifically [7], antidepressants and anxiolytics are recommended based on efficacy evidence in the general population and medically ill patients [8, 9].

Evidence-based clinical guidelines for the management of depression and anxiety in cancer patients have been implemented in Australia [10] and internationally [11]. Although lack of awareness and availability of psycho-oncology services are continuing treatment barriers [12], about 50% of distressed cancer patients decline offers of psychological support [13]. Reasons for declining help include prioritisation of physical disease treatment, preference for self-help, and normalisation of distress [14, 15]. Help-seeking attitudes and behaviour are likely impacted by continuing stigma [16] and low mental health literacy [17] among cancer patients.

Providing patients with knowledge to make informed and values-congruent mental health treatment choices can increase service uptake [18]. Patient decision aids (PDAs) are tools designed to provide evidence-based information and decision support to help patients consider the potential benefits and downsides of available treatment options. PDAs increase patient knowledge and reduce decisional conflict [19] and have been developed for both cancer treatment decisions [20] and mental health treatment decisions in the general population [21, 22]. In oncology settings, where patients are navigating decisions about cancer treatment alongside mental health difficulties, it is critical that they be supported in making values-aligned choices about mental health treatment options. However, there is currently no psycho-oncology specific PD4A. This study aimed to: (1) identify appropriate content and develop a prototype PDA for cancer patients with anxiety and/or depression; and (2) obtain feedback from psycho-oncology professionals and cancer patients/survivors on the perceived accuracy, acceptability, and usability of the PDA.

## Method

### Participants

#### Stakeholder group

Australian psycho-oncology professionals (clinical psychologists and psychiatrists) and academics with expertise in developing decision support tools.

#### Patients

Eligibility criteria were: [1] 18 years of age or older; [2] self-reported diagnosis of cancer within the last 10 years; and [3] sufficient English proficiency.

### Procedure

Development was guided by the International Patient Decision Aids Standards (IPDAS) [23] and the Ottawa Decision Support Framework (ODSF) [24]. The PDA was developed as a booklet for use by patients.

#### Stage 1: Development of prototype PDA

PDA content was based on a literature review and clinical guidelines, with professional graphic design input. The PDA was divided into sections on: [1] the purpose of the PDA, [2] understanding anxiety and depression, [3] psychological and pharmacological treatment options, including risk-benefit information, (making treatment decisions and [5] values clarification exercises (VCEs). Content was supported by info-graphics to convey numerical information and patient quotes.

Patient Education Materials Assessment Tool [25] review yielded an “understandability” score of 94%. See Supplementary File 1 for overview of PDA content. A copy of the IPDAS checklist is provided as supplementary File 2.

#### Stage 2: Alpha testing with clinicians and experts in development of decision support tools

Psycho-oncology professionals were recruited through the Psycho-oncology Co-operative Research Group a national cancer clinical trials professional network of cancer clinicians and researchers working in or with an interest in psycho-oncology. Members with expertise developing decision support tools/resources were purposively invited to participate. Participants were emailed a copy of the PDA for review prior to a Zoom videoconference meeting. Feedback on clinical accuracy, acceptability, and usability was incorporated into the PDA iteratively and presented to clinicians to gain consensus.

#### Stage 3: Alpha testing with patients

Cancer patients/survivors were recruited through email invitation sent to members of Register4, a national online database of people with an experience of cancer interested in cancer research. Participants provided consent prior to completing a short online survey and providing contact details for arranging an interview. A copy of the PDA was emailed to participants prior to interview. Recruitment used a purposive sampling approach and continued until data saturation was achieved.

**Fig. 1:**
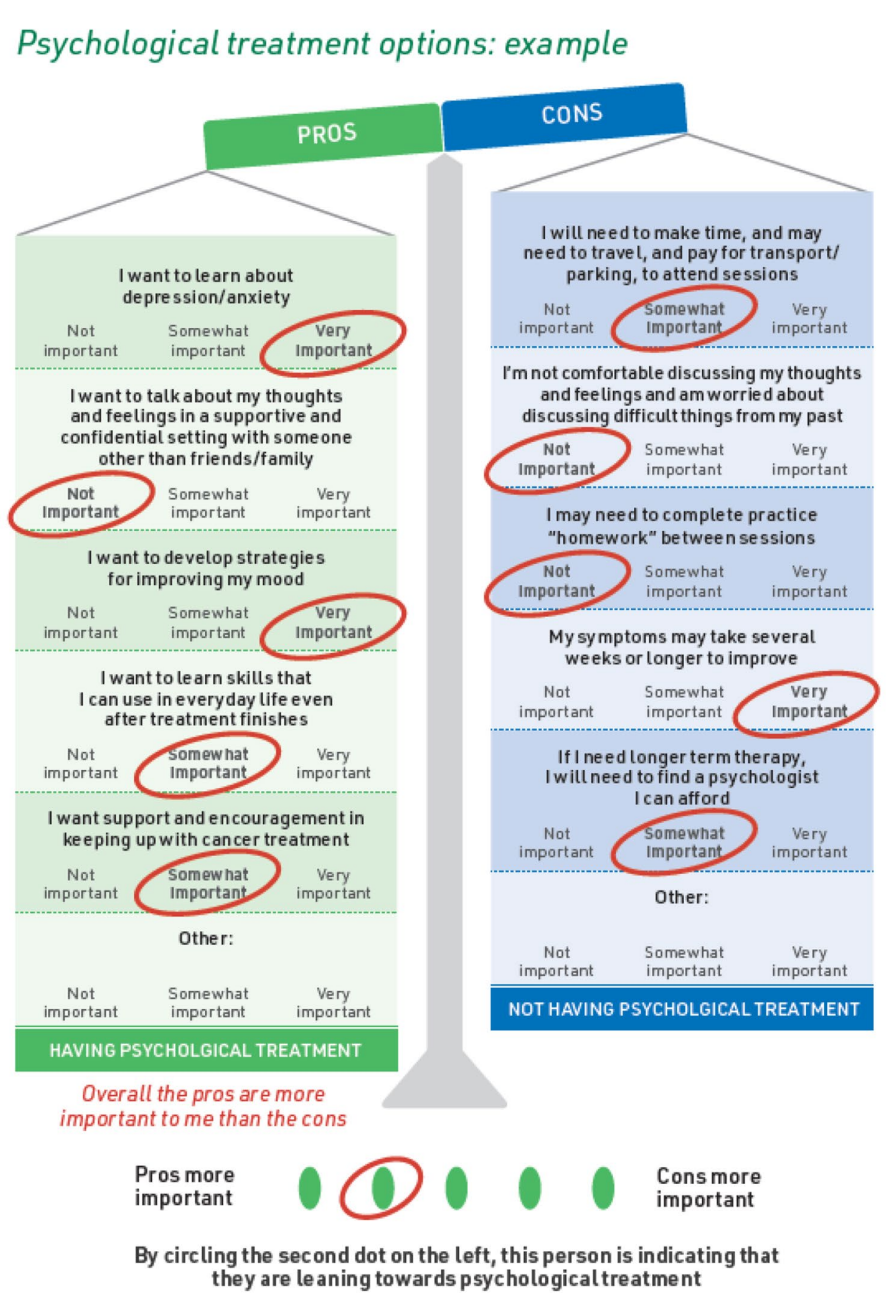
Original values clarification exercise (excerpt)

**Fig. 2:**
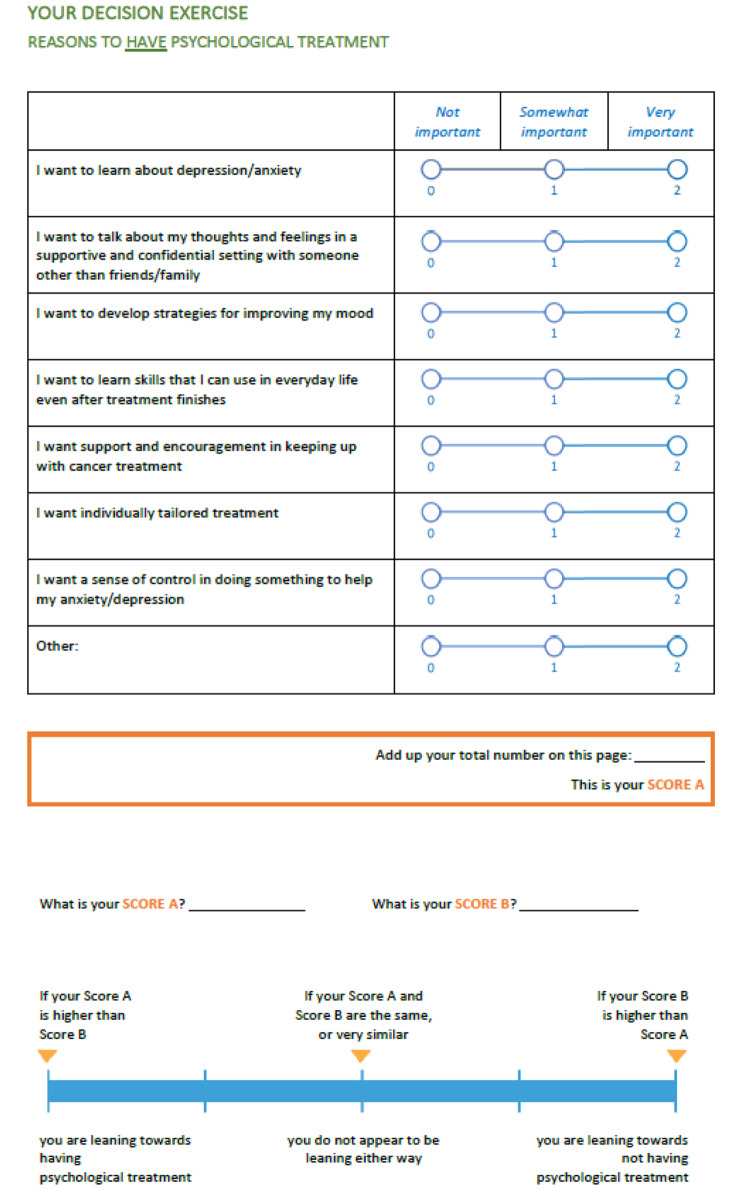
Alternative values clarification exercise (excerpt)

Videoconference (Zoom) cognitive interviews were conducted using ‘think aloud’ [26] methodology and semi-structured interview questions [27] (Supplementary File 3) to obtain feedback on the clarity, ease of use, and perceived usefulness of the PDA. In response to early feedback about clarity of the VCEs (Fig. 1), an alternative format (Fig. 2) was prepared and feedback on both versions sought in subsequent interviews. The second version of the VCEs included a change in format from weighing scales to include explicit descriptors to assist with weighing up the pros and cons and a scoring system based on level of concern (0–2) that could be summated to determine whether patients were leaning towards having or not having a treatment. Interviews were recorded and transcribed verbatim.

### Measures

Demographic and clinical information was assessed using an online (Qualtrics Version 09.2021) survey. An adapted version of the Cassileth Information Styles questionnaire (CISQ) [28] assessed information preferences. Involvement in decision making preferences were assessed using a single-item version of the Control Preferences Scale [29]. The three-item Brief Health Literacy Screener (BHLS) [30] assessed health literacy. Symptoms of depression, anxiety, and stress were assessed using the Depression Anxiety Stress Scales - Short Form (DASS-21) [31].

### Data analyses

Survey data analysis used IBM SPSS Statistics (Version 27). Coding and thematic analysis of interview transcripts using NVivo 12 (QSR International Pty Ltd) was based on Framework Analysis [32]. Two researchers (RR and JS) independently read a selection of transcripts and developed initial codes. After discussion, the researchers agreed on a coding structure which was applied to subsequent transcripts by one researcher (RR) with 20% double coded (JS). The coding structure was refined as required after consensus discussions, and coded transcripts charted into coding matrix charts to compare perspectives across participants and conduct thematic analysis. The Consolidated Criteria for Reporting Qualitative Research (COREQ) [33] guided reporting.

## Results

### Alpha testing with Clinicians and experts in development of decision support tools

Overall, the PDA was perceived as clinically accurate, easy to understand, and potentially usable. Our expert stakeholder panel (*n* = 5) suggested removing detailed information about medication side effects and adding references to self-referral pathways if the PDA was to be publicly available. Minor amendments to text, images, and graphics were recommended to improve clarity and increase engagement. Stakeholders recommended seeking patient feedback on VCE layout.

### Alpha testing with patients

Interviews were conducted with 21 patients/survivors (76% female). Mean interview length was 61 min.

#### Demographics and clinical characteristics

Interview participant mean age was 58.9 years (*SD* = 10.7). Most spoke English at home (90.5%, *n* = 19), were born in Australia (81%) and were tertiary educated (71.5%, *n* = 15). 14.3% (*n* = 3) reported advanced disease at diagnosis and most (95.2%, *n* = 20) had completed their initial treatment. 52.4% (*n* = 11) of participants had previously received, and 38.1% (*n* = 8) were currently accessing, mental health services. Although most participants were psychologically well, 38.1% (*n* = 8) reported clinical depression and 19.0% (*n* = 4) reported clinical anxiety based on DASS-21 responses. Most (85.7%, *n* = 18) participants preferred to receive as much health information as possible. Approximately half (47.6%, *n* = 10) preferred collaborative decision-making, the other half preferring either patient-led (23.8%, *n* = 5) or clinician-led with patient involvement (28.6%, *n* = 6). All participants reported adequate health literacy. See Table 1 for sample characteristics.

**Table 1:** Participant Characteristics

	*n* (%)
**Sample Size**	**21**
**Gender**	
Male	5 (24)
Female	16 (76)
**Age**	
Mean	58.9 (10.7) years
15–25 yrs	0
26–50 yrs	6 (29)
51–75 yrs	15 (71)
>=76 yrs	0
**Highest qualification**	
Year 10 or below (or equivalent)	1 (4.8)
Year 12 / HSC (or equivalent)	0 (0.0)
TAFE certificate/diploma	5 (23.8)
Bachelor’s degree	9 (42.9)
Postgraduate degree (e.g. Masters, PhD)	6 (28.6)
**Current employment**	
Working full-time	6 (28.6)
Working part-time	5 (23.8)
On sick leave	2 (9.5)
Not employed	3 (14.3)
Retired	5 (23.8)
**Country of birth**	
Australia	17 (81.0)
Other (e.g. England, Germany, New Zealand)	4 (19.0)
**Language spoken most at home**	
English	19 (90.5)
Other (e.g. German, Lithuanian)	2 (9.5)
**Ethnicity**	unknown
**Time since cancer diagnosis**	
Less than 12 months	6 (28.6)
1 to 5 years	11 (52.4)
6 to 10 years	4 (19.0)
**Cancer type**	
Breast	7 (33.3)
Gynaecological	1 (4.8)
Breast + gynaecological + liver	1 (4.8)
Breast + lung	1 (4.8)
Lung	1 (4.8)
Melanoma	1 (4.8)
Breast + melanoma	1 (4.8)
Myeloma	1 (4.8)
Breast + Prostate	1 (4.8)
Stomach	1 (4.8)
Thyroid + breast + gynaecological + appendix	1 (4.8)
Bowel + lung	1 (4.8)
Bowel + breast	1 (4.8)
Other (pituitary, carcinoma of ureter)	2 (9.5)
**Cancer stage at diagnosis**	
Localised (Stage 1)	6 (28.6)
Locally advanced (Stage 2 or Stage 3)	7 (33.3)
Metastatic (Stage 4)	3 (14.3)
Unknown	2 (9.5)
Other (“high grade” / cancer spread years after primary diagnosis)	3 (14.3)
**Initial cancer treatment**	
Surgery	3 (14.3)
Surgery + chemotherapy	3 (14.3)
Surgery + radiotherapy	2 (9.5)
Surgery + chemotherapy + radiotherapy	6 (28.6)
Surgery + other ^a^	1 (4.8)
Surgery + chemotherapy + radiotherapy + other ^a^	4 (19.0)
Chemotherapy + radiotherapy + other ^a^	1 (4.8)
Other ^a^	1 (4.8)
**Time since initial treatment completed**	
Less than six months	5 (23.8)
Over six months	15 (71.4)
Have not completed initial treatment	1 (4.8)
**Current treatment**	
Chemo pills	2 (9.5)
Hormone therapy	6 (28.6)
Chemo pills + hormone therapy	1 (4.8)
No other treatments	11 (52.4)
Other (targeted therapy)	1 (4.8)
**Current mental health care**	
GP	1 (4.8)
GP + psychologist	2 (9.5)
Psychologist	4 (19.0)
Psychiatrist	1 (4.8)
None	13 (61.9)
**Previous mental health care**	
GP	1 (4.8)
GP + psychologist	2 (9.5)
Psychologist	4 (19.0)
Psychologist + psychiatrist	2 (9.5)
GP + psychologist + psychiatrist	2 (9.5)
None	10 (47.6)
**Informed about mental health by cancer team**	
Yes	9 (42.9)
No	11 (52.4)
Don’t know	1 (4.8)
**Provided with sufficient information**	
Yes	8 (38.1)
No	2 (9.5)
N/A	11 (52.4)
**Current mood state (DASS-21)**	
Depression	
Normal (0–4)	11 (52.4)
Mild (5–6)	2 (9.5)
Moderate (7–10)	5 (23.8)
Severe (11–13)	2 (9.5)
Extremely severe (14+)	1 (4.8)
Anxiety	
Normal (0–3)	16 (76.2)
Mild (4–5)	1 (4.8)
Moderate (6–7)	1 (4.8)
Severe (8–9)	1 (4.8)
Extremely severe (10+)	2 (9.5)
Stress	
Normal (0–7)	13 (61.9)
Mild (8–9)	4 (19.0)
Moderate (10–12)	1 (4.8)
Severe (13–16)	3 (14.3)
Extremely severe (17+)	0 (0.0)
	*M* (*SD*)
**Information preferences – amount (/5)**	4.62 (0.97)
	***n*** **(%)**
**Information preferences - type**	
As much information as possible	18 (85.7)
Additional information only if good news	1 (4.8)
Only information to take care of myself	2 (9.5)
**Involvement in decision-making**	
Patient-led without clinician	0
Patient-led with clinician	5 (23.8)
Shared/collaborative	10 (47.6)
Clinician led with patient	6 (28.6)
Clinician led without patient	0
**Health literacy**	
Adequate (0–10)	21 (100.0)

Notes: ^a^ Other treatments included stem cell transplant, hormone treatments, immunotherapy, and endocrine therapy

#### Qualitative findings

Participants described the PDA as “*valuable”* [P17], *“informative”* [P13], and *“important”* [P21]. Thematic analysis identified four themes: (1) filling a gap: usefulness of information; (2) ease of use; (3) missing the ‘decision’ in decision aid: misunderstandings of purpose; and (4) supporting decision-making. Themes/subthemes and illustrative quotes are described below.

### Theme 1: filling a gap: usefulness of information

Participants reflected on personal experience of inadequate information about mental health disorders and available treatments. Most reported that the PDA gave them a good understanding anxiety and depression and when treatment might be helpful.

*And for me personally, it would have been really valuable because I didn’t have a clue what to do. [P10]*

*I liked the general explanation around depression as well as anxiety and how there were kind of check points to notice, you know, if you are actually in a deeper state of anxiety or depression or whether that’s on the more normal scale of just day to day fears that you encounter in that situation. So I found that extremely useful. [P21]*

Most participants perceived the PDA provided sufficient information about treatment options, although some noted it did not include information about exercise, support groups or alternative therapies.

*I wondered if it was too detailed at first … But you do want the level, I think you do want the depth of information that’s there … And even if you didn’t sit down the first time and read it all through … I thought the balance was good of not too much information, but certainly enough to be making some decisions about it. [P9]*

*But there were two things that … I thought were critically important in helping me, and they’re not mentioned in the book … exercise … and … groups. [P17]*

While most reported the information on medication options was useful, several participants suggested less detail, preferring to receive information directly from their doctor.

*I don’t think it hurts to know, to have more information [on medication options]. I mean, some people might not use it, but I think it helps to know. [P3]*

*Where they start using the correct terminology [benzodiazepines], I just ignored it … I sort of just depend on the doctors and everybody to inform me about that. [P8]*

Participants generally considered there was a balanced presentation of benefits and downsides of treatment options, although some perceived that downsides visually outweighed the benefits. Some participants expressed concern that making downsides explicit might dissuade people from accessing treatment.

*The downsides are way much heavier optically as the benefits … Just from an optic perspective. Not from the content though. [P21]*

*I just find some people can sometimes get hung up on what the disadvantages are and therefore just close down their receptivity to how it actually might help … you can potentially lose people before you got them. [P9]*

### Theme 2: ease of use

#### Layout and length

Participants described the PDA as *“well set-out”* [P13] and *“easy to use”* [P18]. Most participants reported that checklists and images increased engagement, although several expressed a preference for fewer images and more text per-page. Additional images of young female cancer patients were suggested to increase diversity of representation. Infographics were generally perceived as clear and informative. One participant described feeling *“threatened”* [P21] by the red font, but otherwise fonts and colours were acceptable.

*It’s really visually receptive … Good headings, beautiful colouring. All that sort of stuff makes it very readable. [P9]*

*When you get lots and lots of photos and things like that, to me, it’s just fluff and waffle. [P15]*

Several participants expressed misgivings about the length of the PDA. Nonetheless, there were very few suggestions for removing specific content and participants generally perceived the length to be appropriate for people faced with treatment decisions.

*I guess from my own personal experience, getting another booklet when you’re diagnosed with cancer is a lot, and it was a long, it was a long document … You get so many booklets and then you’re kind of, like, you know, left in a bit of a, I don’t know if I can read another booklet. [P7]*

*At first, I thought it was a bit too much. And then afterwards I thought, Oh no, it’s probably a reasonable amount of information. [P13]*

#### Language: tone and clarity

Participants described the PDA as *“non-threating”* [P9] and *“friendly*” [P4] in tone. Overall, the language was described as *“simple”* [P17], *“easy to read”* [P3], and *“clear”* [P14]. However, some participants queried the meaning of the specific terms (*“feelings of unreality”*; *“management plan”*) and suggested simplifying the descriptions of different psychological therapies. Two participants found the term *“treatment”* too clinical [P5] or triggering [P21], while another stated that *“treatment”* conferred more legitimacy than the alternative term *“therapy”*. Individual participants also objected to the word “*patient*” and the phrase “*cancer care team*”.

### Theme 3: missing the ‘decision´ decision aid: misunderstandings of purpose

Most participants asserted that the PDA, or an abbreviated brochure-style version, should be available to *all* cancer patients at the start of treatment, perceiving it as a reference tool that might support self-diagnosis and self-referral to mental health services.

*I guess with a cancer diagnosis, sometimes people think of the physical straight away … [and] they don’t think about the mental health side of it … So, for me, it would have been a nice tool for the oncologist to have to say … I want you to have this little tool to think about … [because] when your physical health is attacked so much, it is a normal response to an abnormal situation for your mental health to suffer as well. And if you find that that’s happening, please, you know, read this, have this, know that there are options that we can add to support you along the way. [P13]*

When asked when and how they preferred to use the PDA, participants indicated they would prefer to read the PDA in their own time rather than during a consultation with their clinician. Many did not perceive the decision support component as a primary purpose of the PDA and suggested this may need to be more explicitly explained in the introduction.

*I think you had to get a fair way into the booklet to realise that it was about making decisions. Like, there’s a lot of general information about anxiety and depression … the decision-making part of it doesn’t come until quite towards the end. [P18]*

### Theme 4: supporting decision-making

#### Patient quotes

Participants reported that patient quotes broke up the text, normalised anxiety and depression, and increased engagement. Patient testimonials supported decision-making for some participants, but not others.

*One of the things I found really useful in this kind of journey myself is actually speaking to other patients … So being able to see yourself through the quotes of another patient … I think that would be really useful. [P6]*

*I don’t think they help at all. If I imagine me, I think I would make the decision mainly myself and with my family. I wouldn’t care what other people thought. [P14]*

#### Usefulness of VCEs

Most participants perceived the VCEs to be useful. However, a few noted that their pre-existing knowledge about mental health and preference for clinician-led decision-making meant the VCEs were personally less relevant. Two participants also reported they would base their decision-making on *“gut feel”* [P1] and past treatment experience [P17] rather than using the VCEs.

*I’ve never seen anything like that before, so it was really different and really valuable because I’ve never actually sat down and done a pros and cons on treatment before … [In cancer treatment] you’re just told … this is what’s going to happen. And you just have to go with it … So, options is always good. [P10]*

#### Clarity of VCEs

Although most participants reported that the instructions for completing the VCEs were clear, several found the example worksheet *“too complicated”* [P1], *“busy”* [P18], and *“overwhelming”* [P7]. Difficulties completing the VCEs arose in two areas. Several participants found it hard to apply labels assigning relative importance to the downsides (cons) of treatment options. Other participants were confused about how to weigh up the pros and cons to indicate whether they were leaning towards *having* or *not having* a treatment. Several participants suggested an intermediate step was needed where the pros and cons were each tallied.

*[For] the cons, I didn’t know how to say ‘important’ or ‘not important’ … If I needed to make time and travel and pay for it, it wouldn’t have mattered to me … So, I didn’t know whether it was, I had to circle ‘not important’ or ‘very important’ … So, if that didn’t bother me, what would I put down? Not important?... So, the green ones [pros] I answered very easily and quickly, and the cons, I thought about it and thought about it and thought, No, I don’t know. [P8]*

*My question is, How do you score it? … You’ve marked these things up, and then in a second step you get to the bottom … if people are going to put their preferences in, then somehow it needs to be scored at the bottom. [P5]*

Participants highlighted the need for clear instructions given the prevalence of cancer-related cognitive difficulties in addition to symptoms of depression and anxiety.

*People are not necessarily going to be functioning at their best when they’re doing it, not just because they will have a level of anxiety and/or depression … If you’re having chemo, chemo brain is a thing. [P12]*

Most participants shown the alternative VCE format stated it was simpler, clearer, and easier to understand. Participants perceived the layout was less cluttered and it was easier reading across rather than down the page. Conversely, two participants reported that having the pros and cons side-by-side in the original layout was helpful.

*What you’re presenting there is much easier to interpret. It just seems less busy visually and it just, you get that visual analogue scale feeling. It just seems simpler. [P9]*

Participants generally perceived the scoring function would make it easier to determine whether someone was leaning towards having or not having a treatment. However, two participants reported that the score reduced flexibility in decision-making.

*I don’t know, does it give you as much leeway or as much flexibility? I mean, it’s easier for sure, you add them up, and that’s what it is, whereas the other one is a bit like you’re glancing and you’re trying, and then you kind of go, Okay, I think it’s this one, is what I felt, because it’s not definitive yet. [P7]*

Based on participant feedback, revisions to the PDA included: (1) more explicit highlighting of the decision support function on the cover page; (2) removing ambiguous terminology; (3) simplifying the section on medication options; and (4) amending the VCEs to incorporate revised categories and a scoring function.

## Discussion

Increasing uptake of treatment for anxiety and depression among adult cancer patients is critical to patient emotional wellbeing and cancer outcomes. PDAs have been developed to support cancer treatment decision-making and are increasingly being incorporated into mental health decisions. Evidence-based principles based on IPDAS and ODSF frameworks ensure development follows a systematic process of scoping and design, development of a prototype, alpha testing with patients and clinicians and iterative revision prior to broader pilot testing and evaluation [34]. This paper reports on the prototype development, alpha testing, and revision of the first psycho-oncology specific PDA developed internationally. Alpha testing with an expert panel confirmed the PDA was clinically accurate, acceptable, and easy to use. Patients/survivors reported similarly high levels of acceptability and comprehensibility.

PDAs convey complex treatment information in a format that will facilitate greater participation in discussions with the cancer care team. Feedback from participants highlighted the challenge of balancing differing information needs. Several participants raised concerns about the length of the PDA. Misgivings about length were associated with misunderstanding the purpose of the PDA, with several participants suggesting an abbreviated brochure-style document be given to *all* cancer patients at diagnosis. At the same time, participants generally perceived that the PDA presented information about treatment options at a level of detail that would be helpful for patients making treatment decisions. Notably, PDAs of a similar length have previously been used for oncology [35] and mental health [22] treatment decisions and all participants had read the PDA thoroughly prior to interview, suggesting that length was not a barrier to engagement.

Misunderstanding of the purpose of the PDA as a decision support tool is likely due to participants not *actually* making treatment decisions. In this early development phase, participants approached the PDA from the perspective of document review rather than decision-making. Additionally, participants were emailed a PDF rather than hard copy booklet which may have altered how they engaged with the booklet. Perceptions of usefulness were also contextualised within participants’ own decision-making preferences and prior experience, suggesting the PDA was successful in its aim of assisting people to reflect on their personal values. This also supports theoretical arguments that decision support exercises are not useful for all patients and should not be imposed if patients who do not wish to engage in the process [36]. Despite the range of views expressed, overall, most participants perceived the PDA would be useful for people making treatment decisions.

PDAs aim to do more than simply provide information. Key outcomes of PDA implementation are improved decision-making processes and treatment decision quality [19]. Mixed feedback on the clarity and usefulness of the VCE worksheets highlighted the need to amend the exercises. There is currently no best practice for design of VCEs [37]. The balance exercise in the prototype PDA is grounded in decision-making theory [38] and has been effectively used by patients making other mental health treatment decisions [22]. Nonetheless, several participants found this layout confusing. Importantly, one in three cancer survivors may have clinically significant cognitive impairment following chemotherapy [39], underscoring the critical importance of simple VCEs for this patient group. Confusion about how to rate the importance of treatment downsides in our study supports testing alternative wording from other oncology PDAs [35] (“*no benefit/concern*”, “*small benefit/concern*”, “*big benefit/concern*”) along with the scoring function preferred by most participants in our study.

PDAs sit within the broader field of shared decision-making. They are designed to *supplement* discussions with healthcare providers about treatment options by giving patients information to participate in those discussions. Involving expert stakeholders in the development process addresses one barrier to implementation in clinical settings by increasing clinician confidence in PDA content [40]. Most participants in our study preferred to read the PDA alone. However, implementation processes should embed the PDA within a shared decision-making context to ensure the burden of raising mental health concerns is not placed on the patient.

### Study Limitations

This study had several limitations. Patients/survivors were mostly tertiary educated, had current or previous experience of accessing mental health care, were psychologically well, spoke English as their first language, and had adequate health literacy. This potentially limits generalisability of findings regarding acceptability and comprehensibility to patients from other educational, cultural, and linguistic backgrounds or those with clinical anxiety/depression or low health literacy. Participants were also recruited using an registry of people with an experience of cancer who are interested in participating in research. Previous research experience among participants may further limit the generalisability of findings. Additionally, participants in this study were not facing treatment decisions, such that evidence of the PDAs usefulness in improving *actual* decision-making was limited. Further research is needed to pilot the revised PDA with patients who are making treatment decisions to evaluate its effectiveness in improving knowledge and decision quality.

### Clinical implications

Despite the implications of untreated mental health disorders in cancer patients, uptake of referral to psycho-oncology services remains low. Providing patients with information and decision-support to make values-congruent decisions about mental health treatment is vitally important in oncology settings where decision-making is complicated by prioritisation of cancer treatment and normalisation of distress. This study reports on the first steps towards developing a psycho-oncology specific PDA. Further research to confirm the efficacy of the PDA to improve knowledge and assist with values concordant decision making to reduce decisional conflict is required prior to the PDA being implemented broadly in routine cancer care.

## Conclusions

These findings provide valuable feedback on the clarity, acceptability, and usefulness of the PDA. Clinician and patient feedback support the feasibility of developing and implementing a psycho-oncology PDA and highlight the need for comprehensive information and clear and simple decision support for this patient group.

## Electronic supplementary material

Below is the link to the electronic supplementary material.

Supplementary Material 1

Supplementary Material 2

Supplementary Material 3

## Data Availability

Data and materials are available from the authors on request by emailing joanne.shaw@sydney.edu.au.
