# Supplementary material for: Development and user testing of a patient decision aid for cancer patients considering treatment for anxiety or depression
Source: BMC Med Inform Decis Mak. 2023 Apr 6;23:65. doi: 10.1186/s12911-023-02146-y (PMC10080801; doi:10.1186/s12911-023-02146-y)
Supplement: Supplementary file 1 — Supplementary Material 1 [file 12911_2023_2146_MOESM1_ESM.docx]

# Supplementary File 1: Summary of Patient Decision Aid (PDA) Contents

| PDA section | Outline of included content |
| --- | --- |
| General introduction | Purpose of PDA, guidelines for use |
| Anxiety and depression in cancer |  |
| Background | Emotional response to cancer diagnosis  Anxiety/depression vs distress  Implications of untreated mental health disorders |
| What is anxiety? | Definition of anxiety  Common symptoms  When treatment might be helpful  Prevalence information |
| What is depression? | Definition of depression  Common symptoms  When treatment might be helpful  Prevalence information |
| What happens next? | Process for referral/assessment |
|  | Definition of psycho-oncology professionals |
| Treatment options |  |
| Psychological treatment options | Types of psychological treatment  Benefits/downsides  Includes patient quotes about pros/cons |
| Medication options | Types of antidepressants/anxiolytic medication  General information about medication interactions, side effects, and length of treatment  Benefits/downsides  Includes patient quotes about pros/cons |
| What if I don’t take up support? | Pathways for future referral |
| Making treatment decisions | Step-by-step guide for making treatment decisions  Questions to support discussions with a clinician  Family involvement |
| Worksheets: What is important to you about your treatment? | Values clarification exercises (with weight scale visual aids) for psychological and medication treatment options |
| Further resources | Links to Australian-based websites/online resources |
