# Supplementary material for: Development and user testing of a patient decision aid for cancer patients considering treatment for anxiety or depression
Source: BMC Med Inform Decis Mak. 2023 Apr 6;23:65. doi: 10.1186/s12911-023-02146-y (PMC10080801; doi:10.1186/s12911-023-02146-y)
Supplement: Supplementary file 2 — Supplementary Material 2 [file 12911_2023_2146_MOESM2_ESM.docx]

# Supplementary File 3: Interview Guide for Cognitive Interviews

- Introductions, orientation, consent, confidentiality, check PDA review.
- What did you perceive the aim of the booklet is?
- Overall, do you think a booklet like this would be helpful for people trying to decide about treatment options for depression or anxiety?
- Was the booklet easy to use?
- What did you like about it?
- Was there anything you really disliked about it?
- What did you think about the length of the booklet?
- Were there any topics/issues that were not covered in the booklet that would have been helpful to decision making?
- Were there any topics/issues that should be removed from the booklet?
- Was there anything in the booklet that made you stressed or anxious?
- In terms of the presentation of the options, do you think decision aid provided adequate information about all your treatment options?
- Would it have been helpful if a clinician went through some of the pages with you before you looked through the booklet? If so, which ones?
- Would you recommend the decision aid to others?

[Screen share and go through booklet page by page]

- Please look at each section to remind yourself of the content, and as you’re doing this tell me out loud any thoughts that go through your mind. I may also prompt you with some questions as we go through.
- Introductory sections
  - Is it clear what the purpose of the booklet is?
  - How does it feel to you when you open up the booklet?
- Anxiety and depression in cancer
  - Did you perceive the information as helpful?
  - Did it give you an understanding of how anxiety and depression differ from distress?
  - Is there any information that you question?
  - Is anything unclear?
  - Is there anything that was new to you?
  - Is the inclusion of other people’s comments helpful?
- What is anxiety?
  - Did you perceive the information as helpful?
  - Did it provide an understanding of what anxiety is?
  - Did it provide an understanding of when treatment might be helpful?
  - Is anything unclear?
  - Is there anything that was new to you?
  - Is there any information that you question?
  - Is there anything that other people might find confusing?
  - What is your understanding of the infographic?
- What is depression?
  - Did you perceive the information as helpful?
  - Did it provide an understanding of what depression is?
  - Did it provide an understanding of when treatment might be helpful?
  - Is anything unclear?
  - Is there anything that was new to you?
  - Is there any information that you question?
  - Is there anything that other people might find confusing?
  - What is your understanding of the infographic?
- What happens next?
  - Did you perceive the information as helpful?
  - Is anything unclear?
  - Is there anything that other people might find confusing?
- Psychological treatment options:
  - Was the explanation of the different psychological options clear?
  - Is there any information that you question?
  - Is there anything that other people might find confusing?
  - Was the inclusion of other people’s comments on the pros and cons of each option helpful?
- Medication options:
  - Was the explanation of the different medication options clear?
  - Is there any information that you question?
  - Is there anything that other people might find confusing?
  - Was the inclusion of other people’s comments on the pros and cons of each option helpful?
- After the values clarification exercises:
  - Would the exercises help you in deciding about treatment options?
  - If yes, what was helpful?
  - If no, why not?
- Additional prompts:
  - Are you clear about the options available?
  - Were the instructions on how to use the exercises clear?
  - Do the exercises make sense?
  - Is the content clear?
  - Is there anything else that would be relevant to you, in making treatment decisions, that is not captured?
- Alternative VCE format
  - Would this format be helpful in making treatment decisions? Why/why not?
- Conclusion
