# Supplementary material for: Development and user testing of a patient decision aid for cancer patients considering treatment for anxiety or depression
Source: BMC Med Inform Decis Mak. 2023 Apr 6;23:65. doi: 10.1186/s12911-023-02146-y (PMC10080801; doi:10.1186/s12911-023-02146-y)
Supplement: Supplementary file 3 — Supplementary Material 3 [file 12911_2023_2146_MOESM3_ESM.docx]

**Supplementary File 2: International Patient Decision Aids Standards checklist (version 4)**

| **Category** | **Code** | **Item** | **Location** |
| --- | --- | --- | --- |
| **Qualifying** | Q1 | Describes health condition or problem for which index decision is required | **This booklet is designed to help you make decisions about your treatment for anxiety and/or depression PDA page 5** |
|  | Q2 | Explicitly states decision under consideration (index decision) | Psychological and pharmacological treatments described under treatment options PDA pages 22-25 and 28 -29 |
|  | Q3 | Describes the options available for the index decision | Full explanation given in text in section “Treatment Options” 21- 33. |
|  | Q4 | Describes the positive features of each option | Explains potential advantages of both psychological and medication options |
|  | Q5 | Describes the negative features of each option | Explains potential advantages of both psychological and medication options |
|  | Q6 | Describes the features of options to help patients imagine the physical, social and/or psychological effects | Describes what each option would involve and potential advantages and disadvantages; Also included is ‘What happens if I do not take up support?” page 34 |
| **Certification** | C1 | Shows positive and negative features of options with equal detail | Yes, in “Treatment options” section. |

|  | C2 | Provides information about the funding source used for development | n/a as prototype and is unfunded |
| --- | --- | --- | --- |
|  | C3 | Provides citations to the evidence selected | Yes, reference list provided (page 52-55) |
|  | C4 | Provides a production or publication date | n/a |
|  | C5 | Provides information about update policy | n/a |
|  | C6 | Provides information about the level of uncertainty around outcome probabilities | Page 21 under treatment options |
| **Quality** | QA1 | Development included needs assessment to determine what patients need to make the decision | Yes, previous research identified decisional needs. |
|  | QA2 | Development included needs assessment to determine what health professionals need to discuss decision | Yes, previous co-design focus group identified decisional needs. |
|  | QA3 | Development included review by patients not involved in producing the DSI | Yes, this study reports prototype design including consumers |
|  | QA4 | Development included review by professionals not involved in producing the DSI | Yes, this study reports prototype design including psycho-oncology clinicians and patient decision aid expert review |

|  | QA5 | DSI was ﬁeld tested with patients facing the decision | N/A to be evaluated in future study. |
| --- | --- | --- | --- |
|  | QA6 | DSI was ﬁeld tested with practitioners who  counsel patients facing the decision | N/A to be evaluated in future study. |
|  | QA7 | Includes author/developers’ credentials or  qualiﬁcations | n/a to be finalised once the PDA is ready for evaluation |
|  | QA8 | Evidence that DSI improves match between patient preferences and chosen option | N/A to be evaluated in future study. |
|  | QA9 | Evidence that DSI helps patient improve knowledge about options’ features | N/A to be evaluated in future study. |
|  | QA10 | Describes how research evidence was selected/synthesized | Yes, described in methods section of manuscript. |
|  | QA11 | Describes the quality of research evidence used | Yes, described in methods section of manuscript. |
|  | QA12 | Provides step by step way to make decision | Yes, under making treatment decisions page 34-35 |
|  | QA13 | Includes tools to use when discussing options with practitioner | Yes, How do I talk about this with my cancer care team or GP and values clarification exercises 36-43 |

|  | QA14 | Describes the natural course of the condition | Yes under what is anxiety and what is depression sections |
| --- | --- | --- | --- |
|  | QA15 | Makes it possible to compare features of available options | Yes, under advantages and disadvantages section for psychological and medication options |
|  | QA16 | Reports readability levels | PMET score reported in the In manuscript |
|  | QA17 | Provides information about outcome probabilities (OPs) | Not explicitly stated as evidence is not available; severity prevalence data is provided |
|  | QA18 | Speciﬁes reference class of patient for which OPs  apply | Not explicitly stated as evidence is not available; severity prevalence data is provided |
|  | QA19 | Speciﬁes event rates for Ops | Yes, in “what is anxiety and depression sections |
|  | QA20 | Speciﬁes the time period over which OPs apply | n/a |
|  | QA21 | Allows to compare OPs using the same denominator | Yes, |
|  | QA22 | Provides more than one way of viewing probabilities | Yes, text and icon arrays |
|  | QA23 | Asks patients to consider which positive and negative features matter most to them | Yes, in the Making sure your decision reflects your values section pages39-50. |
